# Genome-wide high-throughput screening of interactive bacterial metabolite in the algal population using *Escherichia coli* K-12 Keio collection

**DOI:** 10.1038/s41598-020-67322-w

**Published:** 2020-06-30

**Authors:** Jina Heo, Kichul Cho, Urim Kim, Dae-Hyun Cho, Sora Ko, Quynh-Giao Tran, Yong Jae Lee, Choong-Min Ryu, Hee-Sik Kim

**Affiliations:** 1 Cell Factory Research Center, Korea Research Institute of Bioscience and Biotechnology (KRIBB), Daejeon, 34141 Republic of Korea; 2 Department of Environmental Biotechnology, KRIBB School of Biotechnology, Korea University of Science and Technology (UST), Daejeon, 34113 Republic of Korea; 3 Department of Applied Marine Bioresource Science, National Marine Biodiversity Institute of Korea (MABIK), Seocheon-gun, 33662 Republic of Korea; 4 Molecular Phytobacteriology Laboratory, Infectious Disease Research Center, KRIBB, Daejeon, 34141 Republic of Korea; 5 Department of Biosystems and Bioengineering, KRIBB School of Biotechnology, Korea University of Science and Technology (UST), Daejeon, 34113 Republic of Korea

**Keywords:** Microbiology, Applied microbiology, Microbial communities, Environmental microbiology

## Abstract

Algae-bacteria interaction is one of the main factors underlying the formation of harmful algal blooms (HABs). The aim of this study was to develop a genome-wide high-throughput screening method to identify HAB-influenced specific interactive bacterial metabolites using a comprehensive collection of gene-disrupted *E. coli* K-12 mutants (Keio collection). The screening revealed that a total of 80 gene knockout mutants in *E. coli* K-12 resulted in an approximately 1.5-fold increase in algal growth relative to that in wild-type *E. coli*. Five bacterial genes (*lpxL*, *lpxM*, *kdsC*, *kdsD*, *gmhB*) involved in the lipopolysaccharide (LPS) (or lipooligosaccharide, LOS) biosynthesis were identified from the screen. Relatively lower levels of LPS were detected in these bacteria compared to that in the wild-type. Moreover, the concentration-dependent decrease in microalgal growth after synthetic LPS supplementation indicated that LPS inhibits algal growth. LPS supplementation increased the 2,7-dichlorodihydrofluorescein diacetate fluorescence, as well as the levels of lipid peroxidation-mediated malondialdehyde formation, in a concentration-dependent manner, indicating that oxidative stress can result from LPS supplementation. Furthermore, supplementation with LPS also remarkably reduced the growth of diverse bloom-forming dinoflagellates and green algae. Our findings indicate that the Keio collection-based high-throughput in vitro screening is an effective approach for the identification of interactive bacterial metabolites and related genes.

## Introduction

Aquatic organisms are part of a large ecological network through which many organisms interact with each other throughout their lifetime. In recent decades, a number of studies on the different types of ecological interactions, including mutualism and commensalism, between algae and bacteria have attempted to elucidate a potential industrial application for these interactions^1,2^. These studies have revealed that the algal growth response and physiology can be altered by specific bacteria, and these phenomena lead to interesting industrial benefits, such as algal flocculation and oil accumulation, as well as increased biomass productivity^3–5^. For instance, Cho et al. previously reported that the organic or inorganic carbon exchange in an ecologically-engineered bacterial consortium effectively augmented algal biomass production, alongside improved bioflocculation and cellular lipid accumulation^6^. Furthermore, de-Bashan et al.^7^ demonstrated that bacterial volatile organic compounds (VOCs), such as 2,3-butanediol and acetoin produced by the bacteria *Azospirillum brasilense* and *Bacillus pumilus*, significantly enhanced the growth of *Chlorella sorokiniana*. Similarly, Cho et al.^8^ also reported that the volatile indole content generated by the phycospheric bacteria of the microalga *Chlorella vulgaris* OW-01 promoted increased production of algal biomass and lipid productivity. Finally, Higgins et al.^9,10^ showed that the co-culture of the microalga, *Chlorella minutissima* with the bacterium *Escherichia coli* under mixotrophic conditions effectively enhanced the algal biomass and lipid productivity, in addition to improving the biodiesel quality. Previous studies on bacteria‒microalgae interactions have demonstrated that the growth responses of algae can be altered by various bacterial metabolites, such as indole-3-acetic acid, vitamin B_12_, and bacterial siderophores^2,4,5^. These studies have shown that bacterial metabolites are central regulators of the population and physiology of algae.

The study of bacterial metabolites and their ecological effects not only provide information useful for their industrial use and the study of their ecology, but also for the control of harmful algal blooms (HABs) in aquatic environments. In recent years, climate change has exacerbated the occurrence of HABs, which have become a major ecological issue^11–14^. HABs not only cause severe damage to aquatic ecosystems, but also negatively influence the fishery industry, and can harm human health via bioaccumulation or biomagnification^15–17^. Several strategies have been proposed to control HABs, including chemical treatment—using potassium permanganate and copper sulfate and mechanical control using pumps, barriers, and filters^18–20^. The biological treatment method is an alternative strategy for the control of HABs using organisms, such as algicidal bacteria and viruses^21–23^. For employing the biological method, it is essential to characterize the bacterial interactive metabolites to assess the possible environmental risks and mechanistic reactions. For the identification of bacterial interactive metabolites, we applied a high-throughput screening method using the *E. coli* K-12 mutation library. Two recently developed *E. coli* collections have allowed for the systematic genome-wide search of causative genes that affect the bacteria‒algae interaction^24–26^. The first, the ASKA library, contains *E. coli* cells overexpressing most of the *E. coli* genes from plasmid clones^27^. The second, the Keio collection, includes all the single-gene knockout mutants of all of the non-essential genes in *E. coli* K-12^28^. The aim of genome-wide screening is to identify specific genes associated with a particular phenotype. We hypothesized that the bacterial mutant library could be used to identify causative specific bacterial metabolites affecting the bacteria‒algae interaction. To perform the high-throughput screening of bacterial interactive metabolites, our previous study, we had used a complete set of *E. coli* K-12 ORF archive (ASKA) library, which is a collection of gene over-expressing bacterial cells^26^. Using the ASKA library, we found that the genes related to bacterial riboflavin biosynthesis, namely *ribA*, *ribD*, *ribE*, and *ssuE*, are growth-promoting factors of microalga *C. vulgaris* OW-01. However, the application of a bacterial gene knock-out screening system using the Keio collection was not applied yet. We also hypothesized that the bacterial gene knock-out Keio collection may provide useful information for the mechanistic study of algal growth-inhibitory agents, especially for the screening process. Thus, in the present study, we performed the high-throughput screening of interactive bacterial metabolites and their related genes using the *E. coli* K-12 Keio collection.

## Results and discussion

### High-throughput screening of interactive genes-mediating bacteria-algae interaction using Keio collection

As algae are an essential source of chemical energy for the ecosystem via oxygenic photosynthesis, they are considered a major primary producer of energy. However, eutrophication derived from high anthropogenic nutrient input and altered physical and biological interactions frequently induce the formation of HABs in aquatic environments^29,30^. Bacteria-algae interactions are one of the key mechanisms underlying the formation of algal blooms^31,32^. However, it is highly difficult and time-consuming to verify the bacteria-algae interactive metabolites, especially at the molecular level. To simplify the screening of interactive metabolites, we used the *E. coli* K-12 Keio collection. To verify the algal growth in response to the *E. coli* K-12 Keio collection, a green microalga *C. vulgaris* OW-01, which has shown optimal growth under lab-scale conditions, was used for screening. To specify the most relevant < 100 of algal growth-responsive genes, we have chosen the genes showing above 1.5-fold change than a control. Green microalga *C. vulgaris* OW-01 was effectively applied for our previous screening test using *E. coli* gene over-expression ASKA library^26^. We used the same method of our previous study to evaluate the bacteria-algae interaction using the *E. coli* K-12 Keio collection^26^. The axenic algal culture was confirmed by 18S rRNA sequence analysis and the bacterial colony formation test. To identify the test algal strain, the 18S rRNA was amplified, and the sequence obtained was compared against various sequences of other algal strains using BLAST and Mega software version 7.0^33^. As shown in Supplementary Fig. S1 (A)—based on the partial 18S rRNA-based phylogenetic tree—the isolated strain *C. vulgaris* OW-01 showed a close genetic relationship with *C. vulgaris* KMMCC FC-42 (accession no. HQ702285), *Chlorella* sp. ZB-2014 (accession no. KJ734869), *Chlorella* sp. YACCYB 497 (accession no. MH683919), *C. sorokiniana* (accession no. JX910111), and *Chlorella lewinii* (accession no. FM205861). Moreover, the microscopic image of the isolated strain showed a similar morphology with *C. vulgaris*^34^, confirming that the isolated strain was *C. vulgaris* OW-01 (Supplementary Fig. S1B). As shown in Supplementary Fig. S2A, the axenic condition of *C. vulgaris* was confirmed by electrophoresis of the universal 16S rDNA band. Whereas the rDNA bands were detected in xenic *C. vulgaris* and bacteria culture, the band was not detected in axenic *C. vulgaris* culture. Furthermore, no bacterial cells were detected by microscopic observation in the SYBR green stained samples. These results indicate axenic algal culture was maintained during the experiments.

**Figure 1:**
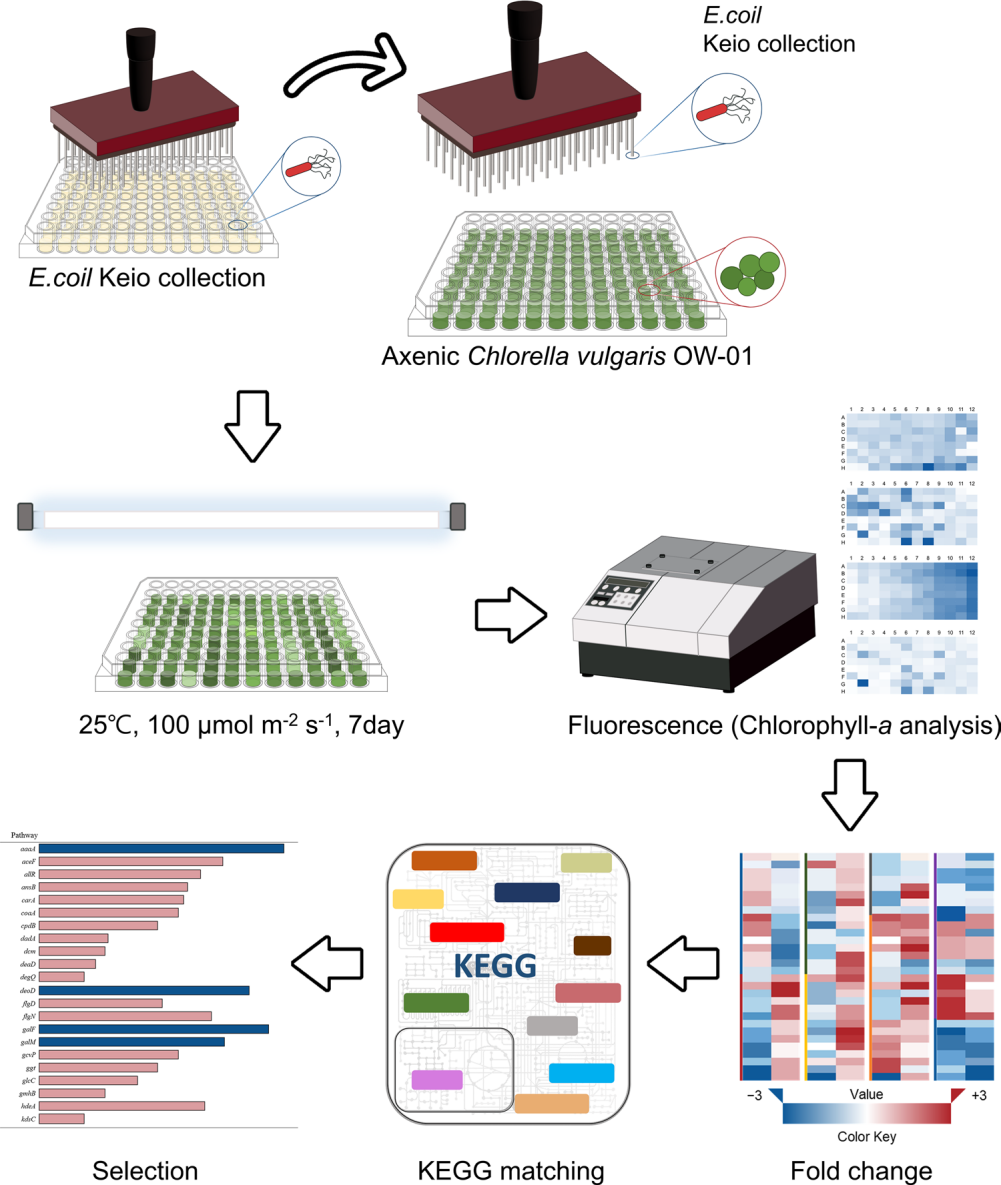
Schematic overview of the high-throughput screening (HTS) for the determination of the bacterial interactive metabolites using the *E. coli* K-12 Keio collection.

After the strain was identified, we tested the *E. coli* Keio mutants using the *C. vulgaris* OW-01 strain to identify the bacteria‒algae interactive genes and metabolites, as shown in Fig. 1. In our preliminary test, the kanamycin supplementation has not affected on algal growth response. Therefore, we supplemented the same concentration of kanamycin into algae-bacterial culture during the screening.

After performing screening three times under optimized algal growth conditions, changes in chlorophyll *a* fluorescence were detected after 7 days of cultivation. By measuring the fluorescence of chlorophyll *a*, we identified bacterial genes of which the absence (deletion) resulted in a 1.5-fold increase in algal growth. The observation of white–yellow color in all screened experimental groups suggests increased population of bacterial cells. However, it is unclear what growth state these bacterial cells were in—due to the lack of available techniques for this purpose. This technical problem should be re-evaluated in further experiments. The Keio collection contains 3,985 gene knock-out mutants and a kanamycin resistance cassette that has been used to create a highly targeted single-gene disruption. Among the 3,985 genes, a total of 80 genes were selected via high-throughput screening, as shown in Supplementary Table S1. The function of each gene is also described in Supplementary Table S1.

The experimental groups showing enhanced microalgal growth (promoted relative fluorescence) were selected as potential genes coding for interactive algal growth inhibitors. To verify the related metabolites, we analyzed the relationships between the selected genes and the bacterial metabolite biosynthesis pathways.

### Role of detected genes in lipopolysaccharide (LPS) biosynthesis pathway

**Figure 2:**
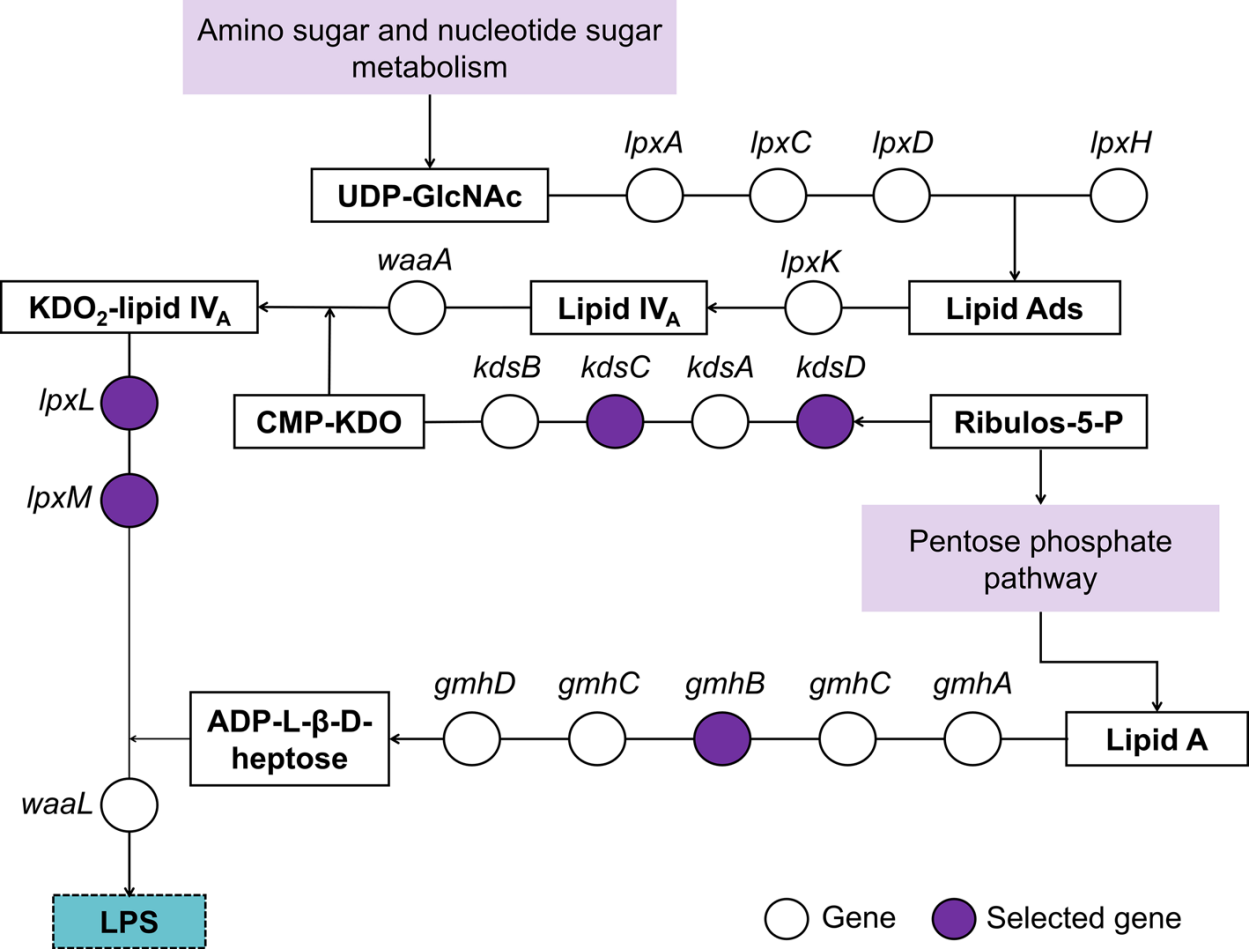
Schematic location of the five selected genes (*lpxL, lpxM, kdsC, kdsD*, and *gmhB*) in the lipopolysaccharide (LPS) metabolism pathway. UDP-GlcNAc, UDP-N-acetylglucosamine; Lipid Ads, Lipid A disaccharide; Lipid IVA, Lipid A disaccharide bisphosphate; KDO2-lipid IVA, Di[3-deoxy-d-manno-octulosonyl]-lipid IV(**A**); CMP-KDO, CMP-3-deoxy-beta-d-manno-octulosonic acid; Ribulose-5-P, Ribulose-5-Phosphate; ADP-l-β-d-heptose, ADP-l-glycero-beta-d-manno-heptose.

As shown in Fig. 2, we investigated the possible associations between related genes in the same pathways. Using the KEGG pathway database and EcoGene 3.0 software, we found that several genes, including *lpxL*, *lpxM*, *kdsC*, *kdsD*, and *gmhB*, were highly associated with LPS biosynthesis. LPS is produced by the amino sugar and nucleotide sugar metabolisms and the pentose phosphate pathway. These pathways synthesize LPS with the help of the detected genes. According to UniProt database (https://www.uniprot.org/), the *lpxL and lpxX* gene encodes lipid A biosynthesis lauroyltransferase and catalyzes the transfer of laurate from lauroyl-acyl carrier protein (ACP) to KDO_2_-lipid IV_A_ to form KDO_2_-(lauroyl)-lipid IV_A_. Furthermore, *kdsC* and *kdsD* code for 3-deoxy-d-manno-octulosonate 8-phosphate phosphatase and catalyze the hydrolysis of 3-deoxy-d-manno-octulosonate 8-phosphate (KDO_8_-P) to 3-deoxy-d-manno-octulosonate (KDO) and inorganic phosphate. The *gmhB* gene encodes d-glycero-d-manno-heptose 1,7-bisphosphate phosphatase, which converts d-glycero-beta-d-manno-heptose 1,7-bisphosphate (beta-HBP) intermediate into d-glycero-beta-d-manno-heptose 1-phosphate by removing the phosphate group at the C-7 position. Ultimately, these genes were closely relevant to the LPS biosynthesis as shown in Fig. 2.

**Figure 3:**
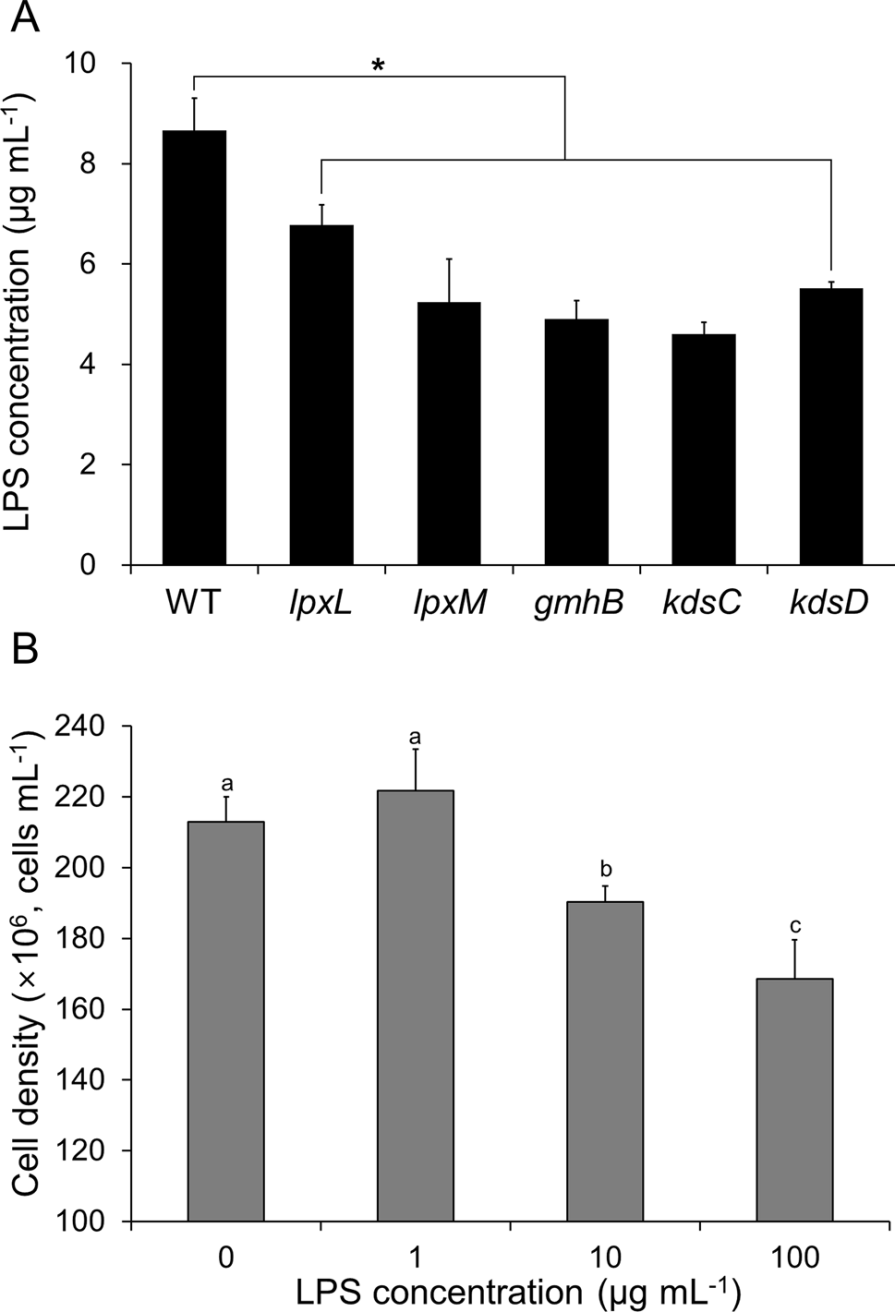
(**A**) Concentration of lipopolysaccharide (LPS) of wild-type (WT), *lpxL, lpxM, gmhB, kdsC*, and *kdsD* mutants cultured in bacterial medium. Error bars represent the mean ± standard deviation (SD) of triplicate experiments. *P ≤ 0.01. (**B**) Effect of LPS supplementation (0–100 µg mL^−1^) on the growth of axenic *Chlorella vulgaris* OW-01. Error bars represent the mean ± standard deviation (SD) from triplicate experiments. Different letters represent significant difference (*P* < 0.05).

To verify the amount of LPS or lipooligosaccharide (LOS) synthesized by the selected mutants, the LPS content was analyzed as previously described (see "Materials and methods" section). As shown in Fig. 3A, all the selected bacterial mutants, including *lpxL*, *lpxM*, *gmhB*, *kdsC*, and *kdsD*, were found to generate significantly lower levels of LPS than the wild-type (about 8.66 μg mL^−1^). Our results indicate that the genes selected by high-throughput screening represent potentially relevant factors for the biosynthesis of bacterial LPS (or LOS). LPS is a well-known gram-negative bacterial endotoxin consisting of a hydrophobic domain lipid A, a core oligosaccharide (COS), and an *O*-polysaccharide (OPS)^35^. LPS has a molecular weight of 100,000 Da with a lipid A portion that is regarded as an in vivo and in vitro endotoxin. Furthermore, the polysaccharide portion of LPS provides immunogenicity, such that the molecular portions stimulate immune responses in cells^36^. The pathophysiological reactions of mammals to LPS, including fever, increased white blood cell counts, disseminated intravascular coagulation, hypotension, and inflammation, have been well-characterized in previous studies and reviews^36–39^. These biological activities stimulate both innate and adaptive immune responses in mammals via the production of cytokines and tumor necrosis factors (TNFs), as well as platelet-activating factors, phagocytosis, and immunoglobulins^36^.

Similarly, LPS also induces immune responses in various terrestrial plants^40^. The immune response of *Arabidopsis thaliana* to OPS-COS isolated from the gram-negative bacterium *Burkholderia cepacia* was reported by Madala et al.^41^. Additionally, Bedini et al.^42^ showed that bacterial LPS, comprised a l-rhamnose-rich backbone, and that synthetic oligorhamnans induce the expression of the pathogenesis-related-1 (*PR-1*) gene and suppress the hypersensitive response in a model of *A. thaliana*. As microalgae can also be classified as thallophytes, we hypothesized that LPS may induce growth and trigger an immune response similar to that observed in plants.

**Figure 4:**
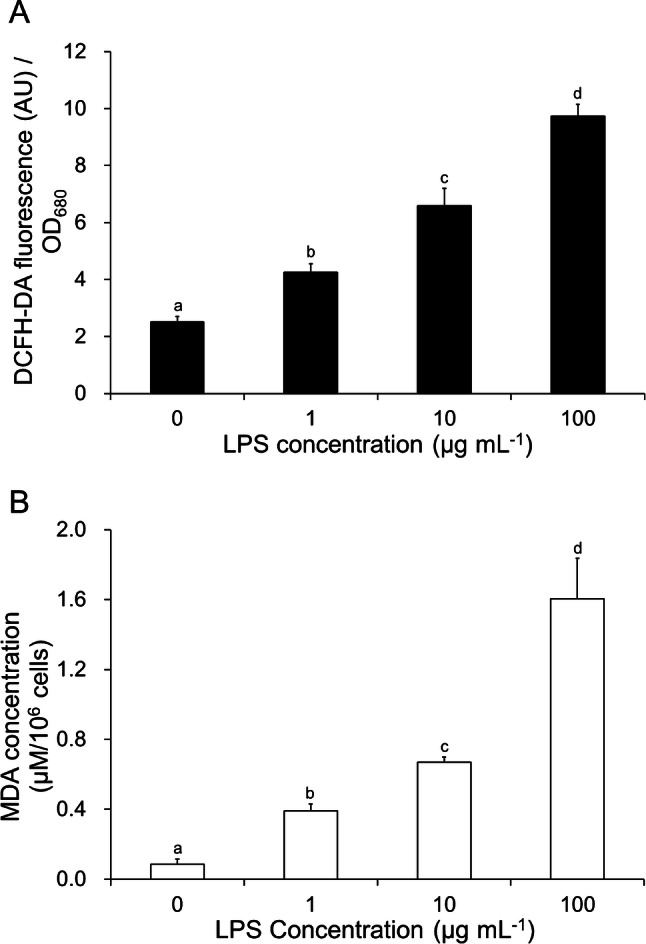
Effect of lipopolysaccharide (LPS) supplementation on the levels of cellular reactive oxygen species (ROS) (**A**) and lipid peroxidation (malondialdehyde content) (MDA) (**B**) of *Chlorella vulgaris* OW-01. ROS was detected by 2,7-dichlorodihydrofluorescein diacetate (DCFH-DA) staining-mediated fluorescence at an excitation wavelength of 485 nm and an emission wavelength of 535 nm. Scale bar, 10 µm. Error bars represent the mean ± standard deviation (SD) from triplicate experiments. Different letters represent significant difference (*P* < 0.05).

To evaluate the growth responses based on the LPS content, the microalga *C. vulgaris* OW-01 was exposed to different concentrations of LPS. As shown in Fig. 3B, algal cell growth was found to be decreased in a concentration-dependent manner when LPS was supplemented at concentrations > 10 µg mL^−1^. These results indicate that bacterial LPS induces a toxic effect in microalgae. To verify the immune response in *C. vulgaris*, we analyzed the changes in the levels of reactive oxygen species (ROS) and the malondialdehyde (MDA) content of LPS-exposed algal cells. As shown in Fig. 4A, the ROS-dependent 2,7-dichlorodihydrofluorescein diacetate (DCFH-DA) fluorescence increased in a concentration-dependent manner when supplemented with LPS at a concentration ranging from 0 to 100 µg mL^−1^. Similarly, the levels of the MDA, which is considered to be a marker for ROS-induced lipid peroxidation, was also enhanced in a concentration-dependent manner upon LPS supplementation, as shown in Fig. 4B. Although there is currently a lack of understanding regarding the role of LPS on the interaction between microalgae and bacteria, our results indicated that the algal growth-inhibitory effect of LPS was highly related to oxidative stress and cellular ROS-mediated lipid peroxidation.

### Effect of LPS supplementation on the growth of diverse algae

**Figure 5:**
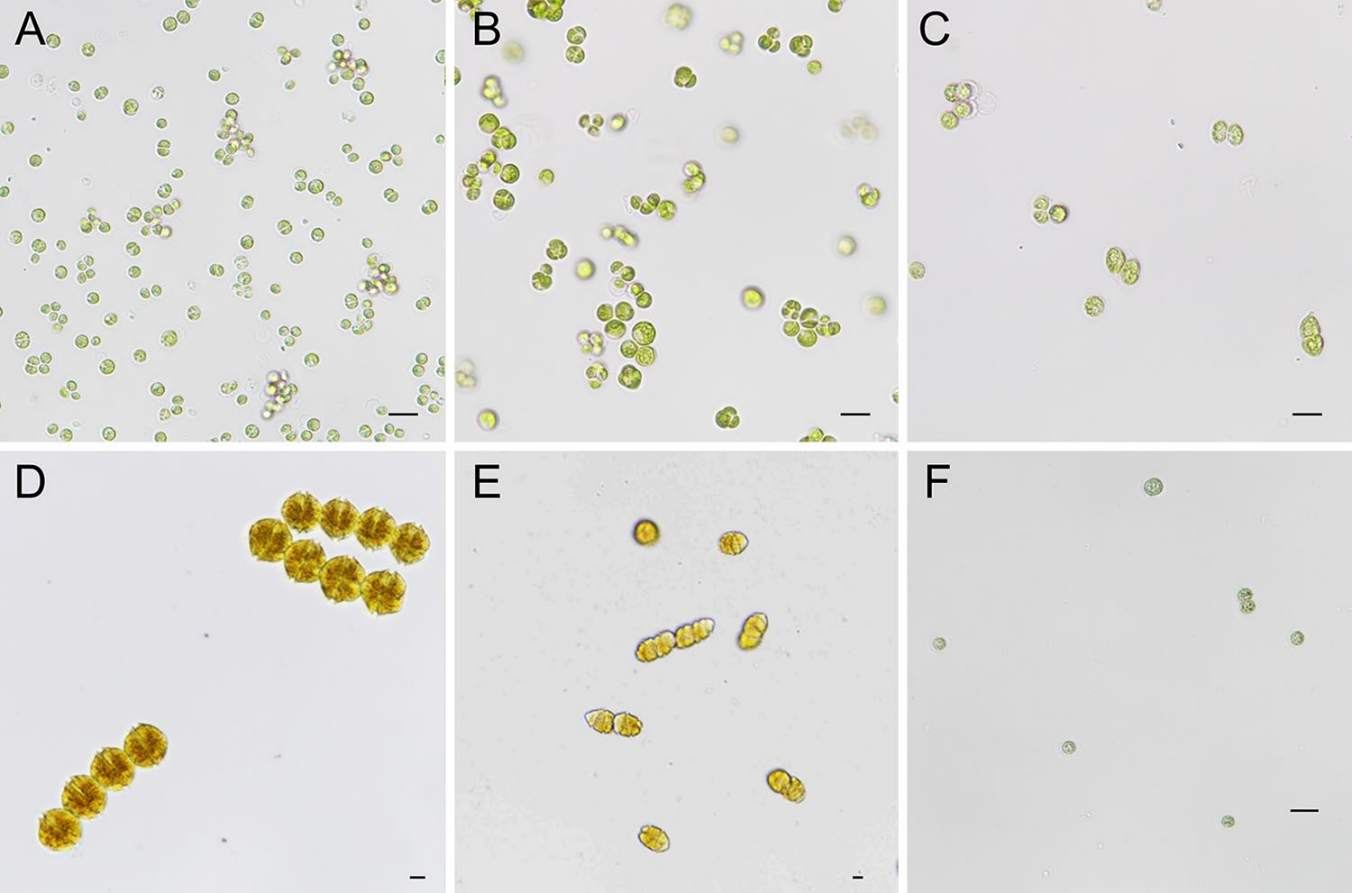
Microscopy images of algal strains. (**A**) *Chlorella sorokiniana*, (**B**) *Parachlorella kessleri*, (**C**) *Scenedesmus deserticola*, (**D**) *Alexandrium tamarense*, (**E**) *Cochlodinium polykricoides*, (**F**) *Microcystis aeruginosa*.

**Figure 6:**
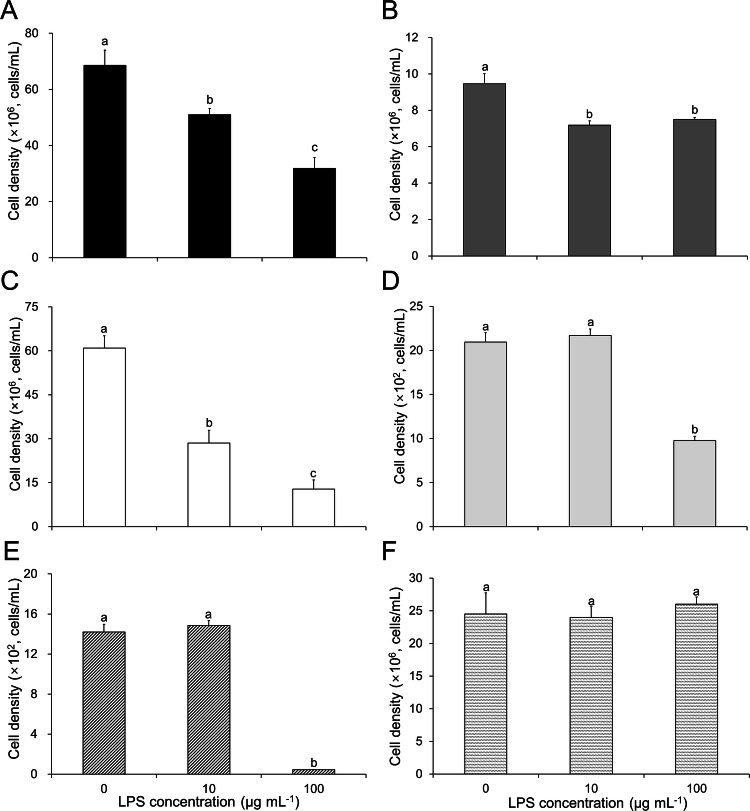
Effect of lipopolysaccharide (LPS) supplementation on the growth responses of various microalgae. (**A**) *Chlorella sorikiniana*, (**B**) *Parachlorella kessleri*, (**C**) *Scenedesmus deserticola*, (**D**) *Alexandrium tamarense*, (**E**) *Cochlodinium polykricoides*, (**F**) *Microcystis aeruginosa*. Error bars represent the mean ± standard deviation (SD) from triplicate experiments. Different letters represent significant difference (*P* < 0.05).

After verifying the algal growth-inhibitory effect and oxidative stress responses resulting from LPS supplementation, we tested the growth responses of other species of algae, including *C. sorokiniana*, *Parachlorella kessleri*, *Scenedesmus deserticola*, *Alexandrium tamarense*, *Cochlodinium polykricoides*, and *Microcystis aeruginosa*. We selected species of microalgae that are important in the industry, including *C. sorokiniana*. *P. kessleri,* and *S. deserticola*, as well as HAB-forming algae, such as *A. tamarense*, *C. polykricoides*, and *M. aeruginosa*, as the test organisms. Microscopic images of the tested algae are provided in Fig. 5, showing the different sizes and morphologies observed via light microscopy. Changes in the cell densities of the algal strains were detected after 7 days of cultivation supplemented with 0, 10, and 100 µg mL^−1^ LPS. As shown in Fig. 6, the majority of the algae tested showed decreased cell growth as a result of LPS supplementation. A concentration-dependent decrease in cell growth was also observed in the green microalgae *C. sorokiniana* and *S. deserticola.* Moreover, the harmful algal species *A. tamarense* and *C. polykricoides* were found to undergo a cell growth-inhibitory effect at an LPS concentration of 100 µg mL^−1^. However, no significant growth response was observed in the cyanobacterium *M. aeruginosa*, as shown in Fig. 6F. Although further mechanistic studies are required, our results indicate that the algal growth response against LPS is species-specific.

HABs caused by *A. tamarense* and *C. polykrikoides* are a well-recognized issue in the global aquaculture industry. The marine dinoflagellate *A. tamarense* is a paralytic shellfish poisoning toxin (PST) that produces harmful marine algae, whose blooms can have serious effects on ecosystems, the aquaculture industry, and public health^43,44^. *C. polykrikoides* is also considered major harmful algae. It is responsible for killing fish by producing reactive oxygen species (ROS), and its bloom has caused serious economic losses in the fishing industry in South Korea (approximately 95.5 million USD dollars in 1995)^45^. To control the formation of HABs generated by these algal species, various biological methods using bacterial species have been developed in recent years^46–52^. To this end, the metabolite-related mechanistic study of the relationship between algae and bacteria can provide useful information for the development of an effective biological strategy for the control of HABs. However, the identification of bacterial interactive metabolites and their related genes is highly difficult and time-consuming. In this study, we performed high-throughput screening using the *E. coli* K-12 Keio mutant collection to demonstrate that LPS is a potential bacteria–microalgae interactive metabolite able to control microalgal growth responses. Our findings indicate that this high-throughput method is a promising method for the effective verification of the bacterial interactive metabolites and related genes involved in the control of HAB formation, as well as algal biomass production.

## Conclusion

In the present work, we developed a high-throughput screening method for the identification of bacteria‒algae interactive metabolites using the *E. coli* K-12 Keio collection. The screening revealed that LPS serves as a possible algal growth-inhibiting bacterial interactive factor via oxidative stress. Furthermore, supplementation with LPS resulted in growth-inhibitory effects on other algal species, including *C. sorokiniana*, *P. kessleri*, *S. deserticola*, *A. tamarense*, and *C. polykricoides.* These results suggest that the *E. coli* K-12 Keio collection-based high-throughput screening can be an effective method for the verification of bacteria–microalgae interactions. However, more in-depth studies, investigating interactions of aquatic ecosystem-relevant bacterial species and bioactivities different types of LPS or LOS, along with the inclusion of each biosynthetic pathway for lipid-A, core and perhaps O-antigen on the algal growth are required to improve the bacteria‒algae interaction mechanism under environmental conditions.

## Materials and methods

### Axenic algal culture

The strain of *C. vulgaris* OW-01 used in this study was previously isolated from a swine wastewater treatment facility in Gongju, South Korea. An axenic microalgal culture was obtained using the micropicking method proposed by Cho et al.^53^. The axenic algal culture was confirmed by colony formation and fluorescence microscopy following SYBR green staining (S9430; Sigma-Aldrich, St. Louis, MO, USA). Further, 16S rRNA was amplified using two universal prokaryotic primers, 27F (5′-AGAGTTTGATCCTG GCTCAG-3′) and 1492R (5′-CGGTTACC TTGTTACGACTT-3′). Gel electrophoresis and sequence analysis were performed to evaluate any bacterial contamination. The obtained sequence data was matched with BLAST (https://blast.ncbi.nlm.nih.gov/Blast.cgi) to confirm the axenic algal culture^54^. The axenic condition was monitored periodically during the experiment. The isolated algal strains were cultivated for 4 days in 50 mL of BG11 medium in a 250 mL Erlenmeyer flask at 25 ± 2 °C under 100 μmol m^−2^ s^−1^ of continuous cool-white fluorescence light, with shaking at 100 rpm^53, 55^.

### High-throughput screening of algal growth response to bacterial metabolites using the Keio collection

The Keio collection is a set of single-gene knockout mutants of the *E. coli* K-12 strain BW25113. This collection contains systematic and gene-deleted bacterial mutants. We compared a total of 3,985 *E. coli* single-cell knockouts lines with *E. coli* K-12 BW25113 serving as the wild-type to screen for the algal growth responses. Before the experiment, the Keio collection was placed in 96-well microplates (Eppendorf, Hamburg, Germany) and stored at − 80 °C after mixing with glycerol (final 40%, w/w). To evaluate the algal growth in response to the knock-out of specific genes in the Keio mutants, a 96-pin microplate replicator (Boekel Scientific, Feasterville PA, USA) was used for the co-culture of bacteria and algae, as shown in Fig. 1. Briefly, the stored glycerol stocks were allowed to thaw at room temperature for 30 min. These were then transferred into a 96-well microplate containing fresh medium and microalgae. For the co-culture of the bacteria and algae, modified algal/bacterial medium (BG11 with 5% Luria–Bertani (LB) media with 50 µg mL^−1^ kanamycin) was used, as proposed by Heo et al.^26^. The initial cell number of the *E. coli* mutants and microalgae was 10^5^ CFU mL^−1^ and 5.0 × 10^6^ cells mL^−1^, respectively. The mixed culture was then stored under algal culture conditions (as described above) for 7 days. For the evaluation of algal growth, we measured the chlorophyll *a*-derived algal auto-fluorescence (excitation of 490 nm and emission 680 nm) using a fluorescence microplate reader (BioTek Instruments Inc., Winooski, VT, USA) after 7 days co-cultivation of bacteria and algae. The chlorophyll *a* readings were normalized by using the axenic control reading as zero [−50 < 0 (axenic control) < 50]. The gene knock-out clones that showed a 1.5-fold increase in relative fluorescence values compared to those of wild-type *E. coli* K-12 BW25113 cells were selected and matched with the Kyoto Encyclopedia of Genes and Genomes (KEGG) pathway database and the EcoGene 3.0 database (https://www.ecogene.org/)^56–58^. To prevent bacterial contamination, all the experimental procedures were performed triplicate using sterilized materials on a clean bench.

### Determination of lipopolysaccharide (LPS) content

To determine the LPS content, the *E. coli* mutants selected in the previous step were pre-cultured overnight in LB medium at 37 °C. Thereafter, the cultured cells (10^5^ CFU mL^−1^) were incubated into 100 mL of fresh BG11 medium containing 5% (v/v) LB medium in a 250-mL Erlenmeyer flask for 7 days. These culture conditions were the same as the co-culture conditions^26^. The mixed culture was then cultivated under 100 µmol m^−2^ s^−1^ of continuous cool-white fluorescence illumination at 25 °C with shaking at 200 rpm for 4 days. The bacterial culture was then centrifuged at 10,000×*g* for 15 min to form cell pellets. The supernatant was used to determine the extracellular LPS content with an abx150357 lipopolysaccharide ELISA Kit (Abbexa, UK), according to the manufacturer’s instructions.

### Analysis of algal growth in LPS-supplemented culture

To verify the results of the Keio collection-based genome-wide screening analysis, the algal growth responses after LPS supplementation were evaluated under lab-scale conditions. *E. coli*-derived powdered LPS was purchased from Sigma-Aldrich (St. Louis, MO, USA) and dissolved in BG11 algal culture medium. Then, the algal growth responses under different concentrations of LPS (0, 1, 10, 100 μg mL^−1^) were analyzed by direct cell counting using a hemocytometer (C-Chip; INCYTO, South Korea) equipped with a Nikon ECLIPSE 80i microscope (Nikon, Tokyo, Japan). The algal culture was performed in a 1,000-mL bottle reactor with 500 mL of the algal medium at an initial cell concentration of 2 × 10^6^ cells mL^−1^. The pre-cultured axenic *C. vulgaris* OW-01 was inoculated into 500 mL of sterile BG11 medium containing different concentrations of LPS and grown at 25 °C with shaking at 200 rpm under a light intensity of 100 µmol m^−2^ s^−1^ for 7 days. To verify the growth responses of other algae, four species of algae, namely *C. sorokiniana*, *P. kessleri*, *S. deserticola*, and *M. aeruginosa*, were isolated from four different freshwater sites (Daejeon, Jeju, Jeonlado, and Cheongju) in South Korea. Two additional algae species, *C. polykrikoides* and *A. tamarense*, were isolated from Tongyeong and Namhae, Republic of Korea. These algal species were then pre-cultured in various media. The freshwater microalgae (*C. sorokiniana*, *P. kessleri*, *S. deserticola*, and *M. aeruginosa*) were cultured in BG11 medium, while the two marine microalgae (*C. polykricoides* and *A. tamarense*) were cultured in F/2 medium^59,60^. All the algal strains were cultured in 500 mL of sterile algal medium in 1,000-mL bottle reactors for 4 days at an optimal temperature (freshwater algae at 25 °C, marine algae at 20 °C). To analyze the algal growth responses, 1 × 10^6^ cells mL^−1^ of *C. sorokiniana*, 1 × 10^6^ cells mL^−1^ of *P. kessleri*, 1 × 10^6^ cells mL^−1^ of *S. deserticola*, 1 × 10^6^ cells mL^−1^ of *M. aeruginosa*, 1 × 10^3^ cells mL^−1^ of *C. polykricoides*, and 1.5 × 10^3^ cells mL^−1^ of *A. tamarense* were treated with 0, 10, and 100 µg mL^−1^ of LPS in each optimal algal culture medium. After culturing, the algal cell density was analyzed using the direct cell method, as described above.

### Detection of reactive oxygen species (ROS) based on 2,7-dichlorodihydrofluorescein diacetate (DCFH-DA) assay

To evaluate the levels of cellular ROS generated after LPS supplementation, LPS-treated algal cells (10 mL) were harvested by centrifugation at 10,000×*g* for 1 min, followed by washing in PBS (pH 7.4) three times. Then, 10 μM of DCFH-DA was added to the algal cell suspension and incubated for 30 min in the dark at 25 °C. The DCFH-DA signals were then measured at an excitation wavelength of 485 nm and an emission wavelength of 535 nm, respectively^61^.

### Determination of lipid peroxidation

The levels of cellular lipid peroxidation were determined using a thiobarbituric acid reactive substance (TBARS) assay to quantify the malondialdehyde (MDA) content. Briefly, 2 mL of algal culture was harvested by centrifugation at 10,000×*g* for 1 min. The resulting pellet was washed twice with PBS (pH 7.4), while the cell suspension was mixed with lysis buffer, comprising 1 μL of 10 mM butylated hydroxytoluene (BHT) solution and 99 μL of radio-immunoprecipitation assay (RIPA) buffer. After bead beating at 4,500×*g* for 1 min, 100 μL of 0.6 N trichloroacetic acid (TCA) solution was added to 100 μL of cell lysate before incubating at room temperature for 20 min. Centrifugation was then performed at 10,000×*g* for 5 min. The resulting supernatant was used for the TBARS assay. The TBARS assay was performed by mixing 150 μL of the sample with 75 μL of 0.5% (w/v) thiobarbituric acid (TBA) reagent. After incubating for 3 h at 50 °C, the absorbance of the culture was measured at 532 nm. The MDA content was measured based on the constructed standard curve of MDA^61^.

### Statistical analysis

The analysis of variance (ANOVA) for experimental data sets was performed using JMP 4.0 software (SAS Institute Inc., Cary, NC). The significant difference was determined based on the magnitude of the *F* value (*P* < 0.01). When a significant *F* value was obtained for a treatment, separation of means was performed by determining Fisher's protected least significant difference (LSD) at *P* < 0.01. The analysis was conducted at least twice, with three replicates per well^26^. One-way ANOVA and subsequent *t*-test were performed to examine significant differences using SPSS 18.0 software (SPSS Inc., Chicago, IL, USA). All the experiments were tested in triplicate.

## Supplementary information

Supplementary information 1

Supplementary information 2
