# Supplementary material for: Genome-wide high-throughput screening of interactive bacterial metabolite in the algal population using Escherichia coli K-12 Keio collection
Source: Sci Rep. 2020 Jun 30;10:10647. doi: 10.1038/s41598-020-67322-w (PMC7327039; doi:10.1038/s41598-020-67322-w)
Supplement: Supplementary file 2 — Supplementary information 2 [file 41598_2020_67322_MOESM2_ESM.pdf]

Genome-wide high-throughput screening of interactive bacterial metabolite in the algal population using *Escherichia coli* K-12 Keio collection

Jina Heo[1,2,a], Kichul Cho[3,a], Urim Kim[1,2], Dae-Hyun Cho[1], So-Ra Ko[1], Quynh-Giao Tran[1,2], Yong Jae Lee[1], Choong-Min Ryu[4,5], Hee-Sik Kim[1,2*]

[1]Cell Factory Research Center, Korea Research Institute of Bioscience and Biotechnology (KRIBB), Daejeon 34141, Republic of Korea

[2]Department of Environmental Biotechnology, KRIBB School of Biotechnology, Korea University of Science and Technology (UST), Daejeon 34113, Republic of Korea

[3]Department of Applied Marine Bioresource Science, National Marine Biodiversity Institute of Korea (MABIK), Seocheon-gun 33662, Republic of Korea

[4]Molecular Phytobacteriology Laboratory, Infectious Disease Research Center, KRIBB, Daejeon 34141, Republic of Korea

[5]Department of Biosystems and Bioengineering, KRIBB School of Biotechnology, Korea University of Science and Technology (UST), Daejeon 34113, Republic of Korea

Running title: Interactive bacterial metabolite screening using Keio collection

[a]These authors contributed equally to this work

[*]**Corresponding author**: Dr. Hee-Sik Kim; Tel. +82-42-860-4326; Fax. +82-42-860-4594; E-mail: hkim@kribb.re.kr

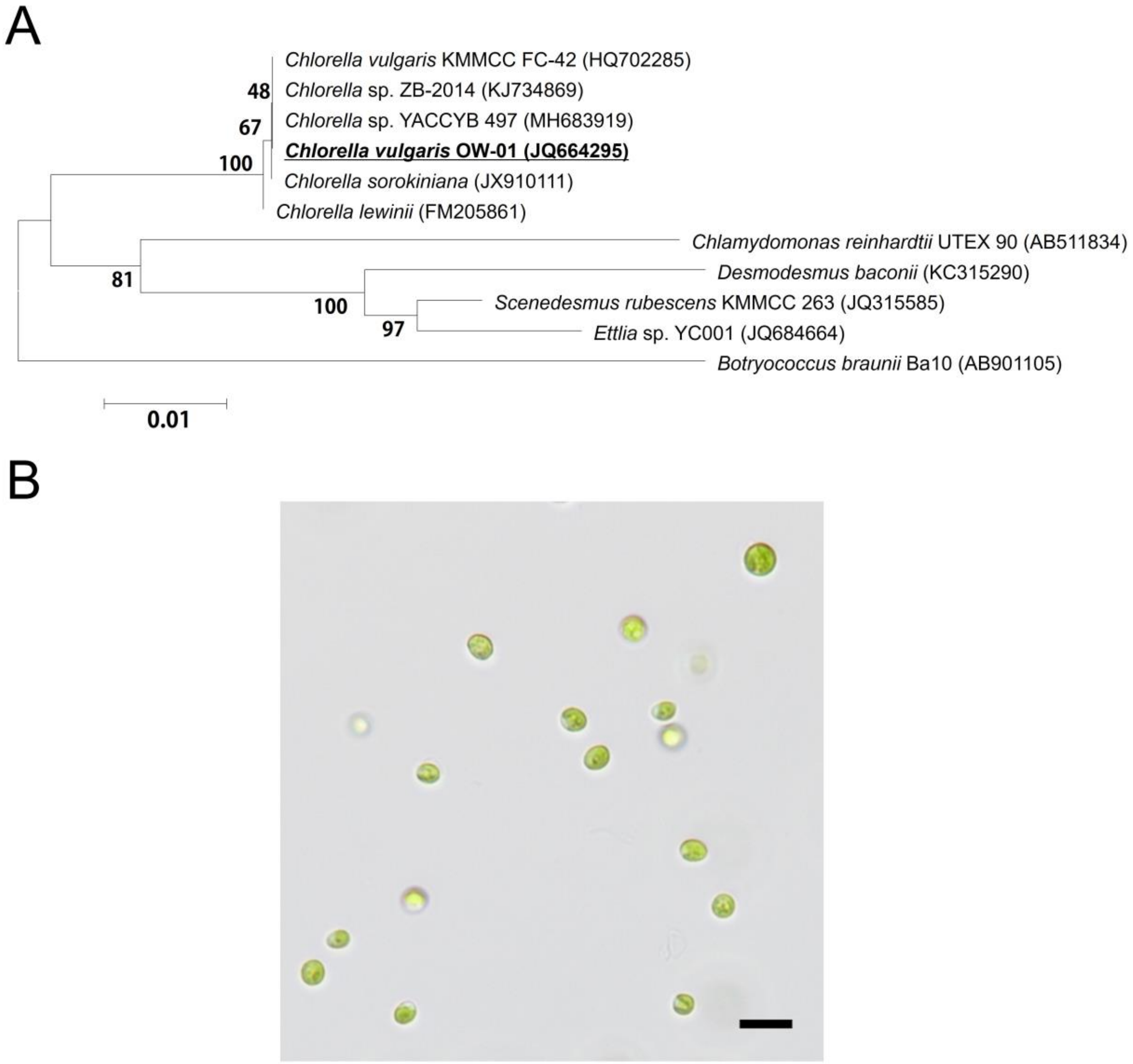

Supplementary Figure S1. (A) Phylogenetic tree of *Chlorella vulgaris* OW-01 (JQ664295) based on the alignment of the partial 18S rRNA sequence. The alignments were performed by the Clustal W program. The tree was subsequently constructed using MEGA7 software. Numbers represent the bootstrap values for 1,000 replicates using the neighbor-joining method. (B) Microscopy image of the isolated strain. Scale bar, 10 µm.

A

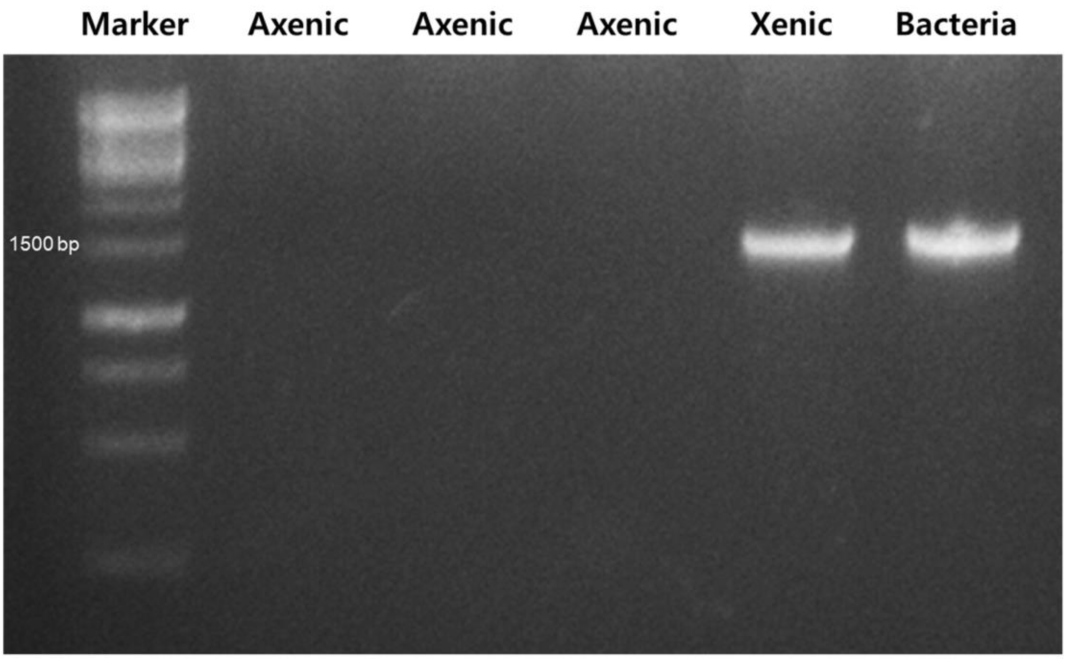

B

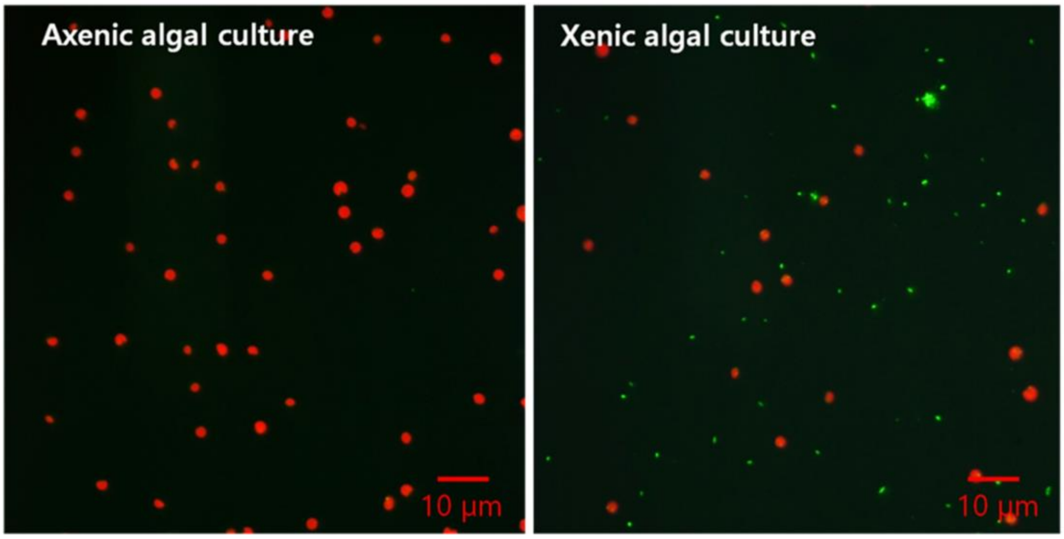

Supplementary Figure S2. (A) The gel electrophoresis of PCR products showing amplified 16S rDNA 1,500 bp gene fragments of the axenic (without bacteria), xenic (with bacteria) algal cultures, and bacterial culture (*Escherichia coli*). For the clarity of axenic condition, triplicate detection was performed using two universal prokaryotic primers; 27F (5'-AGAGTTTGATCCTG GCTCAG-3') and 1492R (5'-CGGTTACC TTGTTACGACTT-3'). (B) Fluorescence microscopic images of axenic and xenic algal culture. Each algal culture was stained with SYBR green. Green fluorescence showing the presence of prokaryotic cells were only detected in the xenic algal culture.

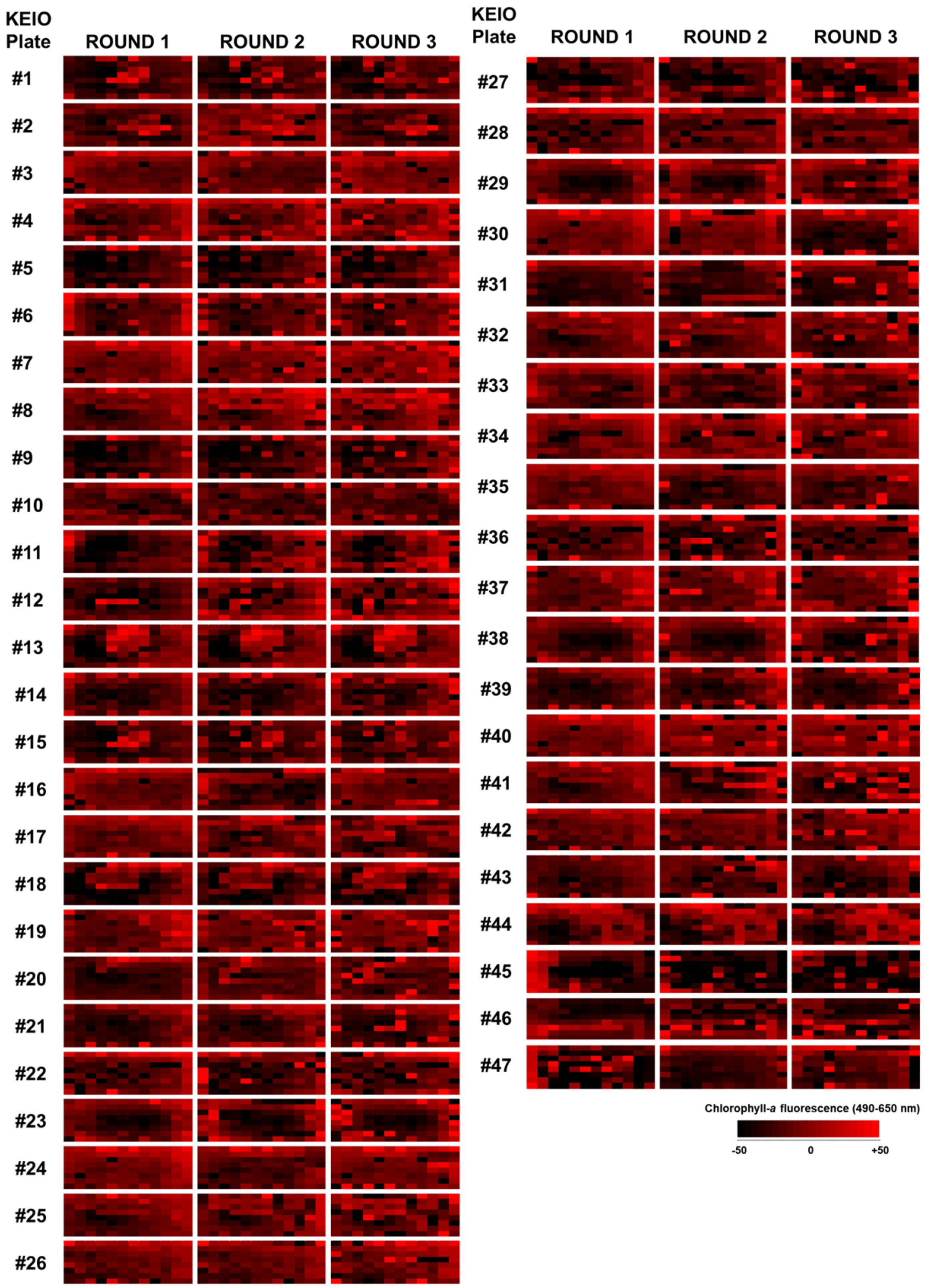

Supplementary Figure S3. The screening of bacterial genes whose expression enhances chlorophyll *a* production in *C. vulgaris*. Heatmap of Chlorophyll *a* content. Red, high chlorophyll *a* content; black, low chlorophyll *a* content.

Supplementary Table S1. The identified bacterial genes showed 1.5-fold increased algal growth than control via *E. coli* K-12 Keio collection-mediated high throughput screening test. The functions of detected genes (*E. coli* K-12 MG1655(U00096.3) were searched via KEGG pathway (www.genome.jp/kegg/pathway.html). Copyright permission of KEGG pathway maps, etc. were obtained via submission of copyright permission request form into the online system (https://www.kegg.jp/feedback/copyright.html).

No.	Identified genes	Gene function[a]
1	*aaeA*	aromatic carboxylic acid efflux pump membrane fusion protein
2	*aceF*	pyruvate dehydrogenase, dihydrolipoyltransacetylase subunit
3	*allR*	DNA-binding transcriptional repressor AllR
4	*ansB*	L-asparaginase 2
5	*carA*	carbamoyl-phosphate synthase small subunit ;glucarate dehydratase;glucarate dehydratase-related protein;MFS transporter, ACS family, glucarate transporter;2-dehydro-3-deoxyglucarate aldolase
6	*coaA*	pantothenate kinase; type I pantothenate kinase
7	*cpdB*	2',3'-cyclic-nucleotide 2'-phosphodiesterase / 3'-nucleotidase
8	*dadA*	D-amino-acid dehydrogenase
9	*dcm*	CMP/dCMP kinase; DNA (cytosine-5)-methyltransferase 1; 5'-deoxynucleotidase
10	*deaD*	adenylyltransferase / [glutamine synthetase]-adenylyl-L-tyrosine phosphorylase; ATP-dependent RNA helicase DeaD
11	*degQ*	serine endoprotease, periplasmic; serine protease DegQ
12	*deoD*	purine-nucleoside phosphorylase
13	*flgD*	flagellar basal-body rod modification protein FlgD
14	*flgN*	flagella synthesis protein FlgN
15	*galF*	UTP--glucose-1-phosphate uridylyltransferase
16	*galM*	aldose 1-epimerase
17	*gcvP*	glycine dehydrogenase
18	*ggt*	YggT family proteinI; gamma-glutamyltranspeptidase
19	*glcC*	GntR family transcriptional regulator, glc operon transcriptional activator
20	*gmhB*	D-glycero-D-manno-heptose 1,7-bisphosphate phosphatase
21	*hdeA*	acid stress chaperone HdeA
22	*hyfI*	hydrogenase-4 component
23	*iadA*	beta-aspartyl-dipeptidase (metallo-type)
24	*kdsC*	3-deoxy-D-manno-octulosonate 8-phosphate phosphatase (KDO 8-P phosphatase)
25	*kdsD*	arabinose-5-phosphate isomerase
26	*lpxL*	Kdo2-lipid IVA lauroyltransferase/acyltransferase
27	*lpxM*	lauroyl-Kdo2-lipid IVA myristoyltransferase

28	*manY*	mannose PTS system EIIC component
29	*metJ*	MetJ family transcriptional regulator, methionine regulon repressor
30	*mrcA*	uncharacterized proteinI; penicillin-binding protein 1A
31	*mutL*	DNA mismatch repair protein MutL
32	*nadB*	L-aspartate oxidase
33	*narK*	MFS transporter, NNP family, nitrate/nitrite transporter
34	*nrdF*	ribonucleoside-diphosphate reductase beta chain
35	*nrfD*	protein NrfD
36	*polB*	DNA polymerase II
37	*priC*	primosomal replication protein N"
38	*prkB*	phosphoribulokinase
39	*purA*	adenylosuccinate synthase
40	*puuR*	HTH-type transcriptional regulator, repressor for puuD
41	*recR*	recombination protein RecR
42	*recT*	recombination protein RecT; DNA-directed RNA polymerase subunit alpha
43	*rfbD*	dTDP-4-dehydrorhamnose reductase
44	*sbcC*	repair protein SbcC/Rad50
45	*sbmC*	DNA gyrase inhibitor
46	*sdhC*	succinate dehydrogenase / fumarate reductase, cytochrome b subunit
47	*seqA*	negative modulator of initiation of replication
48	*sgbH*	3-dehydro-L-gulonate-6-phosphate decarboxylase
49	*smg*	Smg protein; 16S rRNA (guanine527-N7)-methyltransferase
50	*topB*	DNA topoisomerase III
51	*treC*	trehalose-6-phosphate hydrolase
52	*tus*	tRNA 2-thiouridine synthesizing protein E; DNA replication terminus site-binding protein; tRNA-2thiouridine synthesizing protein A, B, C, D
53	*umuC*	DNA polymerase V
54	*uvrB*	excinuclease ABC subunit B
55	*wzxC*	lipopolysaccharide exporter
56	*xdhA*	xanthine dehydrogenase molybdenum-binding subunit
57	*ybiX*	PKHD-type hydroxylase
58	*ycdY*	no KO assigned; (RefSeq) chaperone protein YcdY
59	*ycfT*	no KO assigned; (RefSeq) inner membrane protein YcfT
60	*ycgV*	no KO assigned; (RefSeq) putative autotransporter adhesin YcgV
61	*ychN*	uncharacterized protein involved in oxidation of intracellular sulfur
62	*ycjN*	multiple sugar transport system substrate-binding protein

63	*ycjT*	hypothetical glycosyl hydrolase
64	*ydaV*	putative replication protein
65	*yecC*	ND*
66	*yeeA*	no KO assigned; (RefSeq) putative transporter YeeA
67	*yfaL*	autotransporter family porin
68	*yhbY*	RNA-binding protein
69	*yhgE*	no KO assigned; (RefSeq) putative transport protein YhgE
70	*yhgN*	no KO assigned; (RefSeq) putative inner membrane protein
71	*yhiP*	ND*
72	*yicH*	no KO assigned; (RefSeq) AsmA famly protein YicH
73	*yjfP*	uncharacterized protein; (RefSeq) carboxylesterase
74	*yjhQ*	no KO assigned; (RefSeq) KpLE2 phage-like element; phage-like element, toxin of the TopAI-YjhQ toxin-antitoxin system
75	*yjjI*	no KO assigned; (RefSeq) domain-containing protein YjjI
76	*yjjW*	no KO assigned; (RefSeq) putative glycyl-radical enzyme activating enzyme YjjWno
77	*ymgA*	probable RcsB/C two-component-system connector
78	*yrbL*	no KO assigned; (RefSeq) protein kinase-like domain-containing protein YrbL
79	*ytfJ*	uncharacterized protein; (RefSeq) protein YtfJ
80	*ytfL*	no KO assigned; (RefSeq) putative inner membrane protein

[a]Using the annotation of *E. coli* K-12 MG1655(U00096.3)

*Not detected
